# Portable Solid Phase Micro-Extraction Coupled with Ion Mobility Spectrometry System for On-Site Analysis of Chemical Warfare Agents and Simulants in Water Samples

**DOI:** 10.3390/s141120963

**Published:** 2014-11-06

**Authors:** Liu Yang, Qiang Han, Shuya Cao, Jie Yang, Junchao Yang, Mingyu Ding

**Affiliations:** 1 State Key Laboratory of NBC Protection for Civilian, Beijing 102205, China; E-Mails: 13910919731@139.com (S.C.); yjdyb@163.com (J.Y.); yangjunchao1990@163.com (J.Y.); 2 Beijing Key Laboratory for Microanalytical Methods and Instrumentation, Department of Chemistry, Tsinghua University, Beijing 100084, China; E-Mail: 545-rujia@163.com

**Keywords:** solid phase micro-extraction, ion mobility spectrometry, interface, chemical warfare agents and simulants, sensor

## Abstract

On-site analysis is an efficient approach to facilitate analysis at the location of the system under investigation as it can result in more accurate, more precise and quickly available analytical data. In our work, a novel self-made thermal desorption based interface was fabricated to couple solid-phase microextraction with ion mobility spectrometry for on-site water analysis. The portable interface can be connected with the front-end of an ion mobility spectrometer directly without other modifications. The analytical performance was evaluated via the extraction of chemical warfare agents and simulants in water samples. Several parameters including ionic strength and extraction time have been investigated in detail. The application of the developed method afforded satisfactory recoveries ranging from 72.9% to 114.4% when applied to the analysis of real water samples.

## 1. Introduction

Significant analytical chemistry research efforts are currently aimed at developing suitable analytical methods to facilitate analysis at the location of the system under investigation. This is known as on-site analysis and it is a more efficient approach than laboratory analysis, as is common practice at present. On-site analysis offers many intriguing advantages such as error reduction, elimination of the possibility of sample changes and time delays associated with sample transport and storage, which results in more accurate, more precise and quickly available analytical data. Ion mobility spectrometry (IMS) is a gas-phase electrophoretic technique that allows analytes to be distinguished on the basis of their mass, charge and collision cross section and exhibits instrumental simplicity, low power consumption, real time monitoring, extremely fast response, low operation cost and so on [1–4]. All of these characteristics make IMS feasible for on-site environmental analysis, airport security, safety monitoring and military applications [5–11].

However, a problem that remains is that the sensitivity and selectivity of conventional analytical methods face great challenges for direct measurement of trace analytes in complex samples. With regard to IMS, it makes difficult the characterisation of mixtures, because components present in high concentrations or with a high proton affinity will dominate the spectrum [4]. In addition, matrix components present together with the analyte in the sample may cause problems during analysis. Hence, a sample pretreatment step prior to instrumental analysis is usually necessary. The commonly applied sample preparation techniques, such as liquid-liquid extraction [12,13] and solid-phase extraction [14–16], often require tedious operations and thus are not in favored in on-site sampling and IMS analysis. Solid-phase microextraction (SPME) is a simple sample preparation technique that integrates sampling, extraction, pre-concentration and sample introduction in a single step, which offers the advantages of simplicity, being solvent free, and easy coupling to the subsequent analytical instrument. In particular all these merits recommend the technique for numerous applications in on-site analysis [17]. Many researches have focused on the combination of SPME and IMS by means of simplified modifications of the original SPME sample holder for analysis of volatile organic compounds, explosives, and pharmaceuticals [18–20]. Moreover, the detailed interface exploration was reported to enhance the analytical performance. A thermal desorption SPME inlet constructed from a quartz glass tube and flexible electric heating tape with a hand-held IMS for rapid screening of chemical warfare agent precursors and degradation products in soil was demonstrated [21]. The transfer line/desorber unit which was made up of a low-thermal-mass silicosteel coated stainless steel tube for on-site applications [2]. On the other hand, the problems of the previous researches still remained unsolved. For instance, the IMS instrument was modified to introduce the SPME fiber; the temperature of thermal desorption unit in SPME was non-tunable and this was not beneficial for desorption efficiency as it thus reduced the application scope; furthermore, the power consumption was still relatively high and unfavorable for on-site analysis.

In our work, a novel thermal desorption-based interface was fabricated to integrate SPME with IMS for on-site water analysis. The self-made ultra-portable interface comprised an inner polished stainless steel tube and a low heat mass glass fiber. Chemical warfare agents (CWAs) were selected as the model analytes due to their high toxicity and the use in mass destruction and terrorist attacks, as well as the necessity to detect the presence of such weapons in both military and civil environments. Three CWAs and its two simulants were extracted in water samples and then imported into the IMS for the analysis through thermal desorption. Several parameters influencing the analytical performance were investigated to apply the proposed method to the analysis of real samples.

## 2. Experimental

### 2.1. Chemicals and Standards

All of the chemicals used in the experiment were at least of analytical grade without further purification. Three simulants, including trimethyl phosphate (TMP), triethyl phosphate (TEP) and tripropyl phosphate (TPP), were purchased from Sigma-Aldrich (St. Louis, MO, USA). Two CWAs, including sarin and soman were obtained from State Key Laboratory of NBC Protection for Civilian (Beijing, China). Stock solutions at the appropriate concentration were prepared in methanol, and working solutions were prepared daily by appropriate deionised water dilution of the stock solutions.

### 2.2. Apparatus and Software

The IMS device used in the present study was the handheld Gas Detector Array 2 (GDA2, Airsense Analytics, Schwerin, Germany) based on a 100 MBeq Ni63 ionization source that works in both positive and negative modes. The instrument was switched on and allowed to stabilize for 30 min before measurements began. The operating temperature of the drift tube was 44 °C. The sampling airflow was set at 400 mL(x000B7)min^−1^. The SPME holder with 65 μm PDMS/DVB (polydimethylsiloxane/divinylbenzene) fiber for manual sampling was obtained from Supelco (Bellefonte, PA, USA).

To realize the temperature distribution of the interface, a finite element analysis method using the Steady-State Thermal (SST) module in the ANSYS Workbench software was adopted. This is proven software consisting of tools for implementation of the investigated geometry, for flow simulation and for post-processing of the results. The operation of SST is divided into three stages. First, creation of the model geometry and mesh. According to the flow field, the geometry was generated by a CAD system, and mesh was created using the meshing mode of FLUENT. Second, setting up the solver and physical models, selection of the numerical solver and appropriate physical models, prescribed operating conditions and boundary conditions at all boundary zones. Generation of an initial solution. Setting up solver controls and convergence monitors, and then initializing the flow field. Third, computing and monitoring the solution, examining and saving the results (parameters for the self-made interface: temperature: 250 °C, inner diameter: 2 mm, length: 8 cm; carrier gas: ambient air; parameters for the compared interface [21]: temperature: 200 °C, inner diameter: 5 mm, length: 9 cm; carrier gas: ambient air). The second stage describes and predicts the flow of the carrier gas by solving the coupled differential equations for the conservation of mass, momentum and energy with the help of numerical methods for each particular control volume. In the final stage, the results can be presented graphically (e.g., velocity vector fields, temperature distribution).

### 2.3. Fabrication of the Self-Made Interface

**Figure 1.**
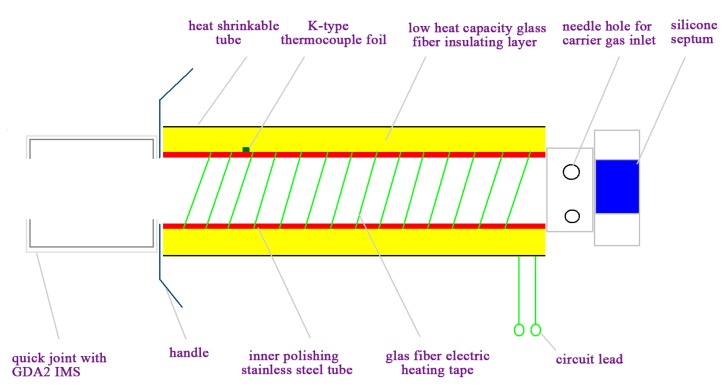
The primary components and constructional details of the self-made interface.

The primary components and construction details of the self-made interface are shown schematically in Figure 1. It consisted of an inner polished stainless steel tube, low heat mass glass fiber insulating layer (thermal conductivity 0.065 w.M/K, Jinan Huolong Refractory materials Co., Ltd., Jinan, China), glass fiber electric resistively heating tape, K-type thermocouple foil (Omega Engineering Inc., Stanford, CT, USA), silicone septum (Beifen Co. Ltd, Beijing, China), needle hole, heat shrinkable tube, proportional integral differential (PID) controller (XMT612, Shenzhen Xinfeiyang Co. Ltd., Shenzhen, China). The inner polished stainless steel tube were wrapped with the glass fiber electric resistive heating tape heating element. Low heat mass glass fiber was wrapped around the stainless steel tube and heating tape to ensure rapid heating. Heat shrinkable tubing was used to insulate and connect the steel tube and other elements. The septum was fitted into one of the caps on the union connector, and ambient air used as carrier gas was introduced via the needle hole. The K-type thermocouple foil used as a fast response sensor connect PID controller could instantly adjust the power supply by comparing the temperature setpoint with the actual temperature. The total length of the SPME-IMS interface device is 12 cm.

### 2.4. SPME Procedures

The extraction were performed by direct immersion (DI) of the SPME device in a 2 mL aqueous standard solution or sample solution, adjusted to the appropriate ion strength, and extraction was for 15 min at ambient temperature (25 °C) without stirring. After extraction, the fiber was inserted into the interface port, piercing through silicone septum into the internal cavity of the interface, depressing the plunger to expose the fiber to thermal desorbtion of the analytes into the IMS detector for analysis.

## 3. Results and Discussion

### 3.1. Evaluation of the Performance of the SPME-IMS Interface

**Figure 2.**
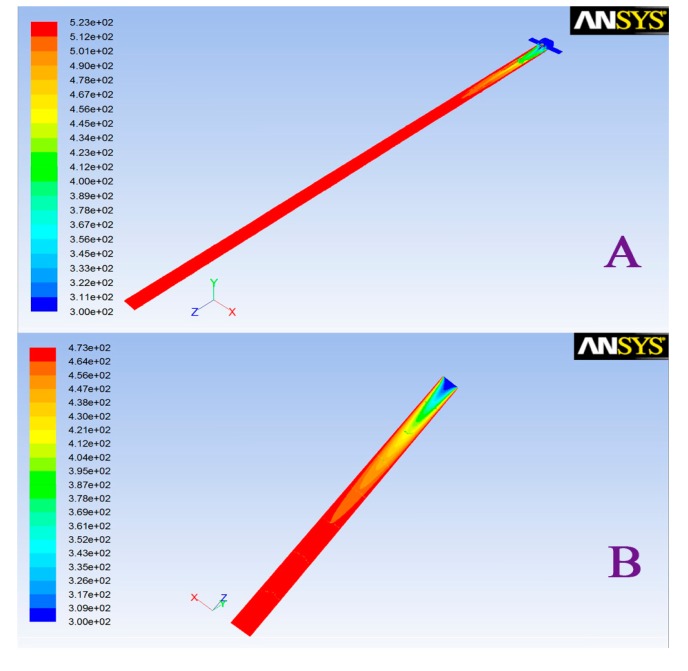
Temperature distribution of the interface designed in our work (**A**) and the published work (**B**). Blue or red colors are presented for the distribution of the different temperature.

Differences in the design of the interface, including inlet structure, tube length and inner radius, have an important influence on its temperature distribution. The traditional completely open gas inlet structure-based interface as reported by Rearden [21], has many problems, such as the depth of the needle may differ in different operations and the time for temperature equilibration is much longer. The interface described in our work adopted a semi-enclosed structure with a chromatography septum gas inlet. Such a design could avoid human disturbance factors caused by different needle shapes and effectively reduce the intake air temperature equilibrium time. To realize the temperature distribution of the interface, a finite element analysis method was adopted for the self-made and the published interfaces using the SST module in the ANSYS Workbench software. The comparison was done under the same inlet carrier gas temperature, different inlet velocity setting, internal tube length, inner radius and carrier gas introduction method, respectively. The result is shown in Figure 2. The interface in our work could achieve temperature balance at a distance of 2 cm from the inlet end compared to 5.1 cm in the other setup. Therefore, the interface designed in this paper can reach temperature balance of the whole internal cavity in a short time, and more quickly realize a uniform internal cavity temperature.

### 3.2. Applications of SPME–IMS System

In order to evaluate the feasibility of the self-made interface for the analysis of CWAs from water samples, the parameters that might affect the performance needed to be optimized.

#### 3.2.1. Reduced Mobility Values of the CAWs

**Figure 3.**
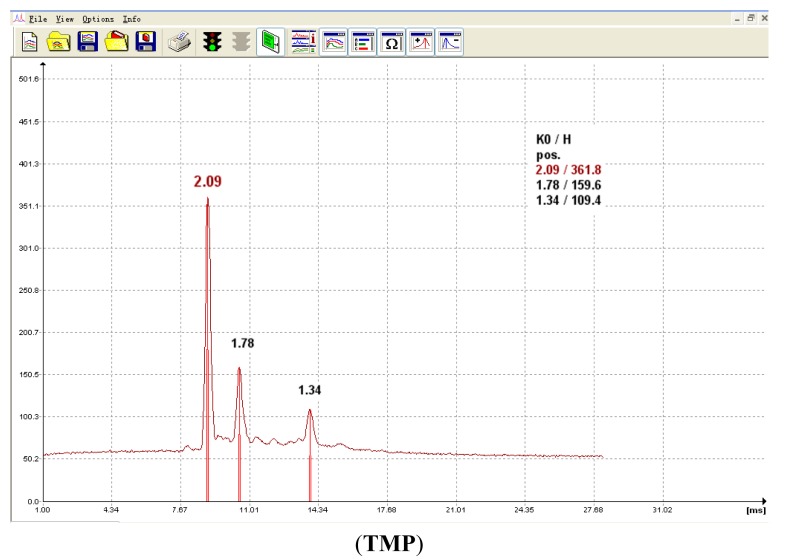

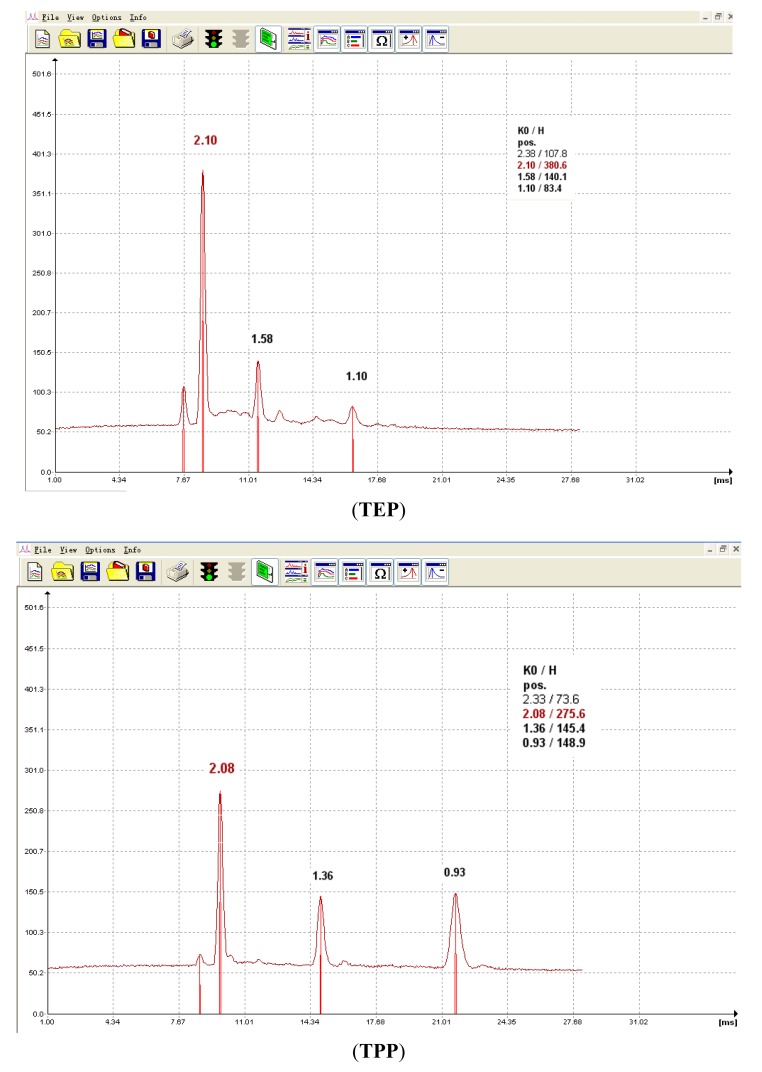
The typical spectra for each CWA obtained though SPME-IMS in water analysis.

In IMS, ion drift times are often reported as reduced mobility constants (K_0_) for identification purposes. This metric provides a basis for comparison of results by correcting for varying environmental and instrumental experimental conditions. In our work, the experimentally determined K_0_ values for each CWA which were obtained in positive mode for each method (including IMS, SPME-IMS and SPME-IMS in water analysis) were consistently matched. The typical product ions for the analytes were their protonated monomer (MH^+^) and dimer (M_2_H^+^) forms and the others were reactant ion peak responses. The K_0_ values of the typical product ions through SPME-IMS in water analysis are listed in Table 1 and the corresponding spectra are presented in Figure 3.

#### 3.2.2. Effect of Ionic Strength

**Figure 4.**
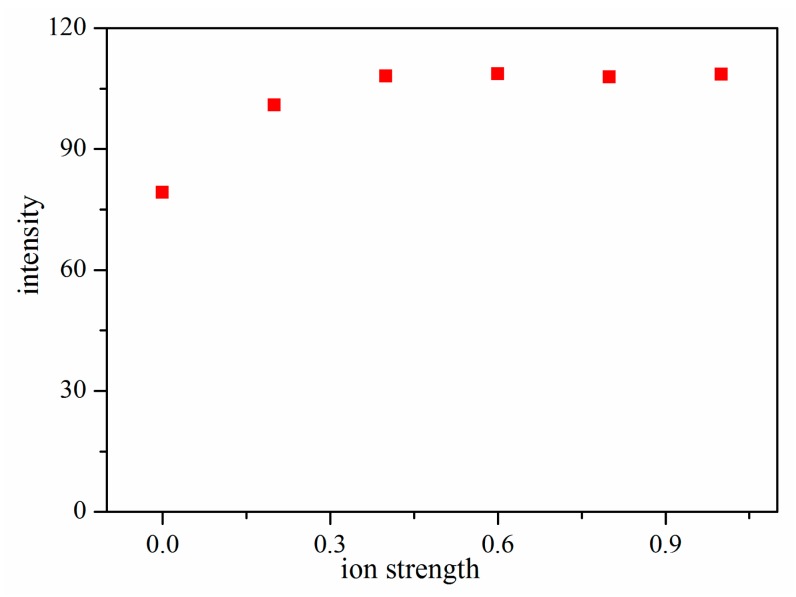
Effect of ion strength on extraction efficiency.

Previous studies have shown that ionic strength can improve the extraction efficiency, particularly for compounds with lower solubility. The ionic strength was modified by adding various amount of NaCl to 5 mL of deionized water. Figure 4 shows that the extraction of TMP increased with the increase of NaCl content, then approached a plateau or even decreased. Therefore, 0.4 g NaCl was added to 5 mL deionized water in the subsequent experiments.

#### 3.2.3. Effect of Extraction Time

**Figure 5.**
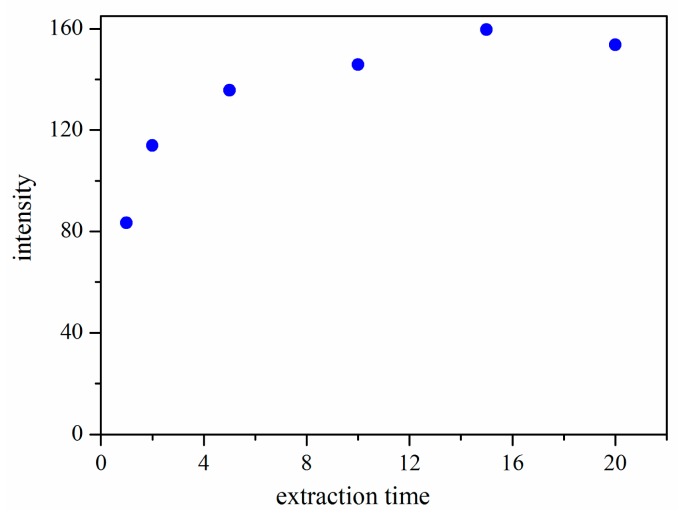
Effect of extraction time on extraction efficiency.

SPME is an equilibrium-based technique, and there is a direct relationship between the extraction amount and the extraction time. In our work, the effect of extraction time on the extraction efficiency of the CWAs was investigated. The extraction time was varied in the range of 0–20 min while other parameters were held constant. All experiments were done in duplicate to ensure reproducibility. The results (Figure 5) indicated that the extraction efficiency increased with the increased extraction time from 1 to 15 min, and then remained almost constant after 15 min. Therefore, the extraction time of 15 min was selected.

### 3.3. Analytical Figures of Merit

The figures of merit for the developed SPME-IMS method for the determination of CWAs are summarized in Table 2. Under the optimized conditions, a series of quantitative parameters with regard to the linear range, correlation coefficient and LOD were examined. The calibration curves of each CWA were constructed by plotting the peak areas (y) of characteristic ions (MH^+^) *versus* the corresponding concentration of the analytes (x). As we know, the linear dynamic range is limited in IMS [22], so we chose the stated range to adapt the method for on-site analysis. The LODs which were calculated as the concentration of the analytes at S/Ns of 2, were in the range of 1.124–1.350 μg/mL. To validate the feasibility of the method, two real water samples were analyzed under the optimized conditions. The results showed that the target CWAs were not found in the samples. To test the accuracy of the method, the recoveries of the method were investigated and the results were in the range of 72.9%–114.4%.

## 4. Concluding Remarks

In this work, a self-made SPME-IMS interface was fabricated. This interface has the advantages of light weight, low power consumption and can be connected directly to an IMS instrument. All of these merits lead to its use in on-site analysis. When applied to the analysis of CAWs in water samples, the developed method obtained satisfactory analytical results.

## Figures and Tables

**Table 1.** The K_0_ values for each CWA obtained though SPME-IMS in water analysis.

**Analytes**	**Typical Product Ions**	

	**MH^+^**	**M_2_H^+^**
TMP	1.78	1.34
TEP	1.58	1.10
TPP	1.36	0.93
Sarin	1.68	1.28
Soman	1.81	1.06

**Table 2.** Parameters of the proposed method for quantitative analysis.

**Analyte**	**Linear Range (**μ**g/mL)**	**Regression Equation**	**R^2^**	**LOD (**μ**g/mL)**
TMP	1.350–2.700	y = 77.4 + 24.4x	0.987	1.350
TEP	1.350–2.332	y = 79.8 + 40.7x	0.989	1.166
TPP	1.350–2.226	y = 64.5 + 25.2x	0.957	1.133
Sarin	1.350–2.464	y = 50.0 + 21.0x	0.957	1.232
Soman	1.350–2.248	y = 63.5 + 30.5x	0.949	1.124

## Glossary

IMS: Ion mobility spectrometry
SPME: solid phase micro-extraction
CWAs: Chemical warfare agents
TMP: trimethyl phosphate
TEP: triethyl phosphate
TPP: tripropyl phosphate
